# Pakistan: A nation held back by NTDs

**DOI:** 10.1371/journal.pntd.0006751

**Published:** 2018-10-18

**Authors:** Alexander J. Blum, M. Farhan Majid, Peter J. Hotez

**Affiliations:** 1 Department of Surgery, Baylor College of Medicine, Houston, Texas, United States of America; 2 Center for Health and Biosciences, James A Baker III Institute of Public Policy, Rice University, Houston, Texas, United States of America; 3 Texas Children’s Center for Vaccine Development, Departments of Pediatrics and Molecular Virology and Microbiology, National School of Tropical Medicine, Baylor College of Medicine, Houston, Texas, United States of America; 4 Center for Medical Ethics, and Health Policy, Baylor College of Medicine, Houston, Texas, United States of America; 5 Department of Biology, Baylor University, Waco, Texas, United States of America; 6 Scowcroft Institute of International Affairs, Bush School of Government and Public Policy, Texas A&M University, College Station, Texas, United States of America; University of Cambridge, UNITED KINGDOM

The Islamic Republic of Pakistan (Pakistan) is the second largest Muslim-majority nation (just behind Indonesia), and with a population of just under 200 million, it’s the fifth most populated country globally [1]. Pakistan’s population is divided into four provinces and three territories (Fig 1). The most populous province is Punjab, which comprises almost one-half of the nation’s population, followed by Sindh, Khyber Pakhtunkhwa (KPK), and Balochistan. The country’s three territories include Gilgit Baltistan, the disputed territory of Azad Jammu and Kashmir, and the Islamabad Capital Territory. About 60% of Pakistanis live in rural areas [2], and 35% of the population is less than 15 years of age [3]. Before and after its independence in 1947, Pakistan has faced high rates of illiteracy, poverty, malnutrition, and disease, all of which have been perpetuated by political instability and conflict.

**Fig 1:**
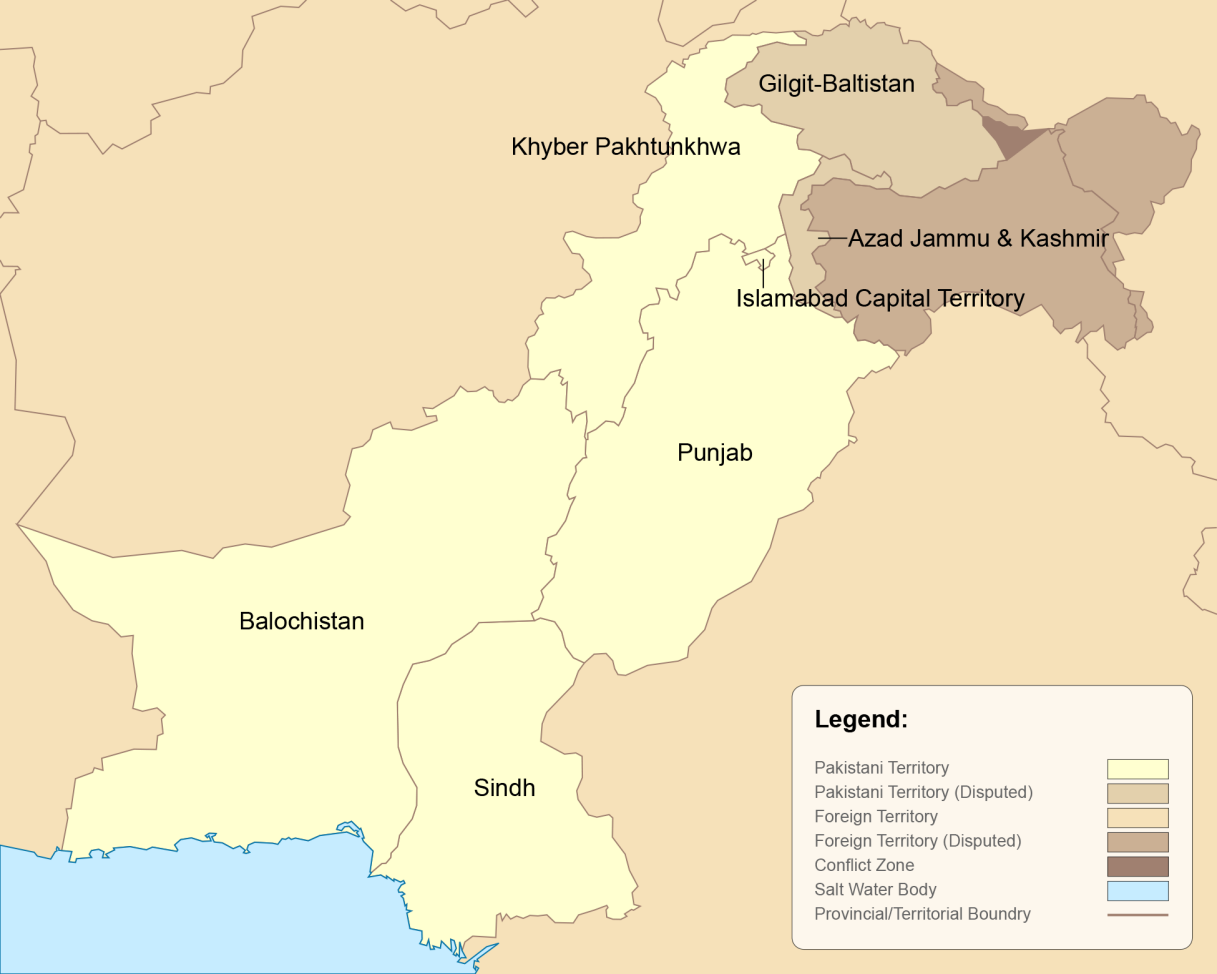
Map of the provinces and federal territories of Pakistan. Image credit: University of Texas PerryCastañeda Library: http://legacy.lib.utexas.edu/maps/pakistan.html.

Despite these obstacles, the country has made some important strides in improving public health.

Pakistan has made innovations in public health delivery, such as a school-based mental-health program [4] and a community-based perinatal and newborn care program [5], which have improved population health in Pakistan and the global health community at large. In terms of vaccines, such delivery mechanisms are perhaps most notably demonstrated by the remarkable decrease in the number of diagnosed polio cases in recent years, which has decreased from 306 in 2014 to just 4 in 2018 to date [6]. Moreover, with the assistance of Gavi, the Vaccine Alliance, Pakistan is expanding vaccine coverage targets for its major childhood diseases, including diphtheria, pertussis, tetanus, hepatitis B, and other diseases [7]. However, severe poverty and poverty-related illness remain widespread. According to the Asian Development Bank, approximately 30% of the population was living below Pakistan’s poverty level in 2013 [8], whereas the World Bank estimates that 6.1% (approximately 12 million people) of the country’s population currently lives on less than US$1.90 per day [1]. Accordingly, the nation still suffers from high rates of infant and under-five child mortality, as well as poor maternal health indicators [8, 9]

In this setting, neglected tropical diseases (NTDs) are widespread, especially among Pakistan’s large rural population but also in many large urban and peri-urban areas [9]. Indeed, an analysis of the Global Burden of Disease (GBD) 2013 study found that Pakistan was one of the top 10 countries in terms of the highest absolute number of NTD cases for 8 of 18 NTDs on which statistics had been gathered [10]. Shown in Table 1 are the latest estimates for either prevalence or incidence of Pakistan’s major NTDs (as well as malaria, for comparison) as determined by the GBD 2016 [11]. Overall, ascariasis and other soil-transmitted helminth (STH) infections, as well as dengue and venomous animal contact (snake bite), now represent the most common NTDs, but typhoid and paratyphoid fevers, leishmaniasis, trachoma, and leprosy are also prominent.

**Table 1:** Pakistan’s major NTDs. Data from GBD 2016 [11].

Disease	Prevalence^a^ or incidence^b^ in 1990 (% population^c^)	Prevalence or incidence in 2016 (% population^d^)	Change between 1990 and 2016 (% change based on population affected)
Ascariasis	13.74 million (12.8%)	25.33 million (13.1%)	+84% (+03%)
**Dengue**	**0.17 million (0.2%)**	**2.57 million (1.3%)**	**+1,412% (+744%)**
Hookworm	1.24 million (1.2%)	2.37 million (1.2%)	+91% (+07%)
Trichuriasis	1.04 million (1.0%)	1.98 million (1.0%)	+90% (+06%)
**Venomous animal contact**	**0.96 million (0.9%)**	**1.95 million (1.0%)**	**+103% (+13%)**
**Malaria**	**3.70 million (3.4%)**	**0.98 million (0.5%)**	**−74% (−85%)**
**Typhoid**	**0.68 million (0.6%)**	**0.57 million (0.3%)**	**−16% (−53%)**
Cutaneous leishmaniasis	0.34 million (0.3%)	0.42 million (0.2%)	+24% (−31%)
**Paratyphoid**	**0.27 million (0.3%)**	**0.29 million (0.2%)**	**+07% (−40%)**
Blinding trachoma	0.19 million (0.2%)	0.10 million (0.1%)	−47% (−71%)
Cystic echinococcosis	22,401 (<0.1%)	13,724 (<0.1%)	−39% (−66%)
Leprosy	5,061 (<0.1%)	4,973 (<0.1%)	−02% (−45%)
**Rabies**	**2,549 (<0.1%)**	**928 (<0.1%)**	**−64% (−80%)**

^a^Nonbolded indicates prevalence.

^b^Bolded indicates incidence.

^c^From 107.6 million (https://www.populationpyramid.net/pakistan/1990/).

^d^From 192.8 million (https://www.populationpyramid.net/pakistan/2016/).

**Abbreviations:** GBD, Global Burden of Disease; NTD, neglected tropical disease.

## Parasitic NTDs

According to WHO, approximately 32 million Pakistani children require periodic deworming for their major STH infections [12], but it is unclear what portion of these children actually receives regular anthelminthic treatments. Indeed, WHO points out that Pakistan has the highest burden of STH infections in its Eastern Mediterranean region [13], and it’s interesting to note that the share of Pakistan’s population with ascariasis, hookworm, or trichuriasis has not changed since 1990, according to the GBD. Among the three STH infections, ascariasis stands out for its high prevalence in selected areas of Pakistan; based on the 2016 GBD data, 13.1% of the country’s citizens are infected with roundworm. This abundance is consistent with the parasite’s known ability to better tolerate conditions with hotter temperatures and higher aridity—found across much of Pakistan—and to thrive in urban and rural environments alike [14, 15]. Overall, Pakistan’s burdens of STH infections are not equally distributed across all of the country’s communities and locales. According to a recent survey from schools nationwide in Pakistan, the highest prevalence occurs around northern regions of Punjab [16, 17], with Rawalpindi and Gujrat representing the areas of highest prevalence (56% and 31%, respectively). STH infections are also endemic in the northern province of KPK, with highest prevalence around the district of Swat (27%), near the borders of Afghanistan [17], with the highest prevalence districts representing “large populous cities” [17]. Another epidemiological survey that focused on Swat found that over 70% of the 1,041 stool samples collected were positive for infection with one or more STH species [18]. Of these, 60% were infected with Ascaris worms, and people working as shepherds were found to be the most vulnerable population [18]. Much of the southern region of Pakistan exhibits low levels of infection, with the notable exception of the Karachi area, where prevalence reaches 20% [17]. In Pakistan, hookworm infections can be found in both urban and peri-urban settings [19, 20], so as its major cities expand, we can expect urban hookworm infection to emerge as a major NTD in Pakistan. Following the three intestinal nematode infections, cystic echinococcosis is perhaps the next most widespread helminthiasis in Pakistan, as it is across central Asia [21, 22].

Pakistan’s burden of cutaneous leishmaniasis (CL)—which exceeded 400,000 cases in 2016—accounts for almost 10% of all cases globally. Both *Leishmania tropica* and *L*. *major* CL are endemic, although *L*. *tropica* has emerged as the major pathogen among internally displaced individuals fleeing military operations against terrorist strongholds in Waziristan [23]. It’s believed that the numbers of CL cases in Waziristan are increasing dramatically, as they are elsewhere in other war-affected areas of Afghanistan and the Middle East [23]. Despite the strong social stigma of disfiguring lesions [24], access to essential medicines and other CL therapies remains limited in Pakistan [25, 26].

## Viral, bacterial, and other NTDs

*Aedes aegypti*, the major mosquito vector of dengue, has emerged in Pakistan, especially in the northern and southern regions where dengue is now widespread [27]. *A*. *albopictus* is also present, and in some areas, both vector species are found and are capable of transmitting dengue and potentially other arboviruses [28]. According to the GBD 2016, the incidence of dengue has increased more than 1,000% since the 1990s when the first major outbreak occurred in Karachi [29], such that it is now the second most common NTD behind ascariasis. The largest dengue outbreaks occurred in Lahore in 2011, which resulted in more than 300 deaths, and then in Swat in 2013 with more than 50 deaths [29]. Dengue outbreaks and epidemics are most common following monsoon season, with dengue virus (DENV) 2 and DENV 3 often predominating in urban areas [29]. Chikungunya has also emerged as an important arbovirus infection [30]. Rabies remains an important viral NTD in Pakistan. Its persistence is due in part to failure to administer proper post-exposure measures—this includes using home remedies instead of thoroughly washing wounds—and is compounded by lack of access to vaccines and rabies immune globulin [31]. Moreover, an obsolete vaccine made from nerve tissue is still produced in Pakistan, which in some cases has inadequate or zero potency [31].

Among the bacterial NTDs, typhoid and paratyphoid fevers remain endemic in Pakistan, where they also represent major causes of mortality. With respect to the former, *Salmonella enterica* serovar Typhi has emerged as an extensively drug resistant (XDR) bacterial species during an epidemic in Sindh, Pakistan [32], and there are concerns that this strain could spread globally [33]. Trachoma also remains endemic, and Pakistan has the highest disease burden of leprosy in the WHO Eastern Mediterranean region [34]. With regards to the former, the International Trachoma Initiative (ITI) has partnered with the Pakistan Ministry of Health to begin mass drug administration with azithromycin [35]. For Hansen’s disease (leprosy), the ready availability of curative drugs makes it an appealing target for widespread targeted multidrug therapy through existing treatment networks in Pakistan. The most expansive of these are led by Aid to Leprosy Patients (ALP) based in Rawalpindi, and Maria Adelaide Leprosy Centre (MALC) based in Karachi [34]. Finally, snake envenomation represents an important noninfectious NTD in Pakistan, associated with significant morbidity and mortality, especially in rural and mountainous areas [36].

## Concluding comments and future directions

The prevalence rates of Pakistan’s most common debilitating NTDs, including STH and other helminth infections, cutaneous leishmaniasis, trachoma, and leprosy, do not appear to have undergone significant changes over the last 25 years. Moreover, since 1990, dengue and XDR typhoid fever have emerged as concerning public health issues. Although there is little overall work in terms of an economic impact assessment of NTDs in Pakistan, based on existing evidence from other low- and middle-income countries (LMICs), we expect that large sections of Pakistani children and adults are trapped in poverty due to the impact of these conditions on productive capacity, child development, and pregnancy outcomes [37]. NTDs may also account for the high rates of child malnutrition found in Pakistan [38]. There is a notable concentration of NTDs, including STH infections, in Pakistan’s urban centers, and it has been predicted that in the coming decades we can increasingly expect to find NTDs emerging in urban and peri-urban settings of expanding megacities in developing countries [39].

The Government of Pakistan and its Federal Ministry of Health have had some important public health successes in terms of polio elimination and reductions in other vaccine-preventable childhood diseases, but there is a need to add NTD reductions to these victories. Regarding STH infections, WHO notes that a mapping survey is being completed in collaboration with nongovernmental partners, including the Deworm the World initiative of Evidence Action [13]. For trachoma, prevalence surveys have been conducted since 2007, which was expanded to 15 districts in 2015 [40]. In 2017, mass drug administration for trachoma (together with the Surgery, Antibiotics, Facial cleanliness, Environmental improvement [SAFE] strategy) was implemented in in several districts in collaboration with ITI [40]. Additional reports indicate that the Federal Ministry of Health (in collaboration with WHO) has expanded its vector control activities in response to dengue and other epidemics, but seasonal floods and other crises have hampered these efforts [41].

In some cases, there is also a need for new biotechnologies, possibly including an improved rabies vaccine, and new vaccines for Pakistan’s emerging health threats, such as XDR typhoid fever, dengue, and urbanized hookworm infection. Pakistan has well-developed scientific infrastructures, which has successfully produced nuclear technologies, but so far this is not fully translating towards biotechnologies [42]. There are urgent needs to expand both implementation science and biotechnology in Pakistan so it can better assess, treat, and prevent its widespread NTDs. Vaccine and science diplomacy represents an important yet mostly untapped resource for capacity building in the country [43].
